# Differences in Dietary-Related Experiences, Determinants, and Information-Seeking Behaviors Among Pregnant and Postpartum Women: Cross-Sectional Survey Study

**DOI:** 10.2196/73808

**Published:** 2026-09-28

**Authors:** Janine P M Faessen, Desiree A Lucassen, Edith J M Feskens, Elske M Brouwer-Brolsma

**Affiliations:** 1 Department of Human Nutrition and Health, Wageningen University & Research, Stippeneng 4, Wageningen, 6708 WE, The Netherlands, 31 (0) 317 - 480080

**Keywords:** pregnancy, postpartum, nutrition, dietary support, maternal diet, mobile health, mHealth, apps

## Abstract

**Background:**

Multiple studies have highlighted the prevalence of suboptimal dietary patterns during pregnancy and the postpartum period, with some studies indicating factors that influence dietary behaviors.

**Objective:**

This study aimed to map the differences in dietary-related experiences, determinants, and information-seeking behaviors, focusing on app use, during the pregnancy and postpartum periods among Dutch women.

**Methods:**

In total, 1619 women recruited via social media filled in an online questionnaire consisting of 32 multiple-choice, 2 ranking, 11 rating, and 20 open-ended questions covering demographics, dietary-related activities during pregnancy and the postpartum period, determinants of these activities, and information-seeking behaviors focusing on app use. Questions were specific to pregnancy or postpartum, and responses were compared using chi-square tests, ANOVA, or 2-tailed *t* tests between perinatal phases.

**Results:**

We obtained data from 842 pregnant and 777 postpartum women. Mean age was 32 (SD 4) years, mean self-reported prepregnancy BMI was 25 (SD 5) kg/m², and most participants were highly educated (1214/1619, 75.0%). Pregnant and postpartum women valued healthy nutrition (mean 8.5, SD 1.2 on a 10-point scale). Pregnant women rated their food enjoyment and diet quality lowest during the first trimester, with scores of 6.3 (SD 2.5) and 6.3 (SD 1.9) out of 10, respectively. Compared with pregnant women, postpartum women had less phase-specific nutritional knowledge (354/745, 47.5% vs 533/820, 65.0%), less time for food preparation (365/726, 50.3% vs 618/803, 77.0%), and less time for eating (524/721, 72.7% vs 773/799, 96.7%), but were more eager to learn about phase-specific healthy food choices (313/745, 42.0% vs 234/820, 28.5%) and felt more capable of food preparation (mean 7.3 SD 2.2 vs mean 6.9, SD 2.2). During pregnancy, the main reason for eating was the child’s health, while in postpartum, the main reason was hunger. Key sources of nutrition information were websites (1337/1565, 85.4%), health care professionals (805/1565, 51.4%), and apps (665/1565, 42.5%). Phase-specific app use dropped from 90.8% (713/785) during pregnancy to 58.9% (418/710) postpartum, with many women being unaware of the existence of postpartum apps. Important factors to consider for app use included credibility, ease of use, and free access.

**Conclusions:**

Our results highlighted significant differences in dietary-related experiences, determinants, and information-seeking behaviors between pregnant and postpartum women, underscoring the need for tailored dietary support tools for each phase. The limited availability of postpartum nutrition resources, such as apps, highlighted the need to develop improved digital nutritional counseling solutions.

## Introduction

A healthy diet during pregnancy and the first year postpartum is critical for maternal and child health, for example, lowering risks of gestational diabetes, suboptimal gestational weight gain, gestational hypertension, preeclampsia, low birth weight, and postpartum depression [1-8]. Suboptimal diets are common among pregnant and postpartum women in high-income countries [1,9], characterized by insufficient intakes of folate, iron, vitamin D, and fiber [10,11], along with increased intakes of total and saturated fats [12]. Regarding food groups, these dietary patterns generally align with a noticeable decline in fruit and vegetable intake and increased discretionary food consumption [12].

Women encounter both facilitators and barriers that shape healthy eating during pregnancy and the postpartum period, and these influences may vary across different phases. During pregnancy, motivation to prioritize the baby’s health is typically strong, but it can vary along with the capability to eat healthily throughout different stages of pregnancy [12]. Qualitative studies in the United Kingdom indicated that physical discomfort in the first trimester may impede healthy eating, whereas in midpregnancy, some women may prioritize a nutritious diet while others may prioritize enjoying their meals again, contrasting with their experience earlier in pregnancy [13,14]. A Canadian study found that adherence to a high-protein and high-dairy intervention decreased from the first to the last trimester of pregnancy, indicating potential changes in motivation or physical complaints [15]. Focusing on the postpartum period, which is mainly concentrated on adaptation and physical recovery, a recent review among women in the United States indicated that the postpartum period presents additional challenges including emotional eating, physical limitations, social support deficits, food environment constraints, childcare needs, time constraints, and the prioritization of maternal responsibilities [16,17]. While postpartum women did consider their child as a facilitator for healthy eating, they found it difficult when experiencing limited time and energy [17].

The perinatal period, marked by increased health care engagement and driven by mothers’ strong desire for optimal child health, presents a crucial opportunity to encourage healthier eating habits [1,9,12]. Nonetheless, access to high-quality dietary advice and support from health care professionals (HCPs) seems limited in high-income countries [18,19], including the Netherlands [20-22]. Dutch HCPs report difficulties with providing extensive dietary support because of a lack of knowledge and time [20,21]. As a result, women frequently search online for pregnancy- and postpartum-specific information, with a general focus on infant health [23-25]. Pregnancy-specific nutrition information is often sought online because of convenience and limited support from HCPs [26], whereas searching for maternal postpartum nutrition information appears to be very limited [23]. Additionally, the use of mobile health (mHealth) apps is rapidly expanding [27-29] with a notable presence of pregnancy-specific apps [22,29]. However, the available apps in Dutch and Australian app stores lack expert involvement, tailored support, and consistency with guidelines [22,30].

Improving dietary support during the perinatal period is important to promote healthy maternal eating and benefit both maternal and child health. To effectively improve such support, it is important to understand women’s dietary-related experiences, determinants, and information-seeking behaviors during pregnancy and the postpartum period, as well as how these may differ between and within these phases. Therefore, the aim of this questionnaire study was to determine phase-specific and within-phase differences in dietary-related experiences, determinants, and information-seeking behaviors, focusing on app use, between pregnant and postpartum women living in the Netherlands. With additional questions on information retrieval and app use, the results of this study can inform user-centered mHealth development for personalized dietary support during pregnancy and postpartum period.

## Methods

### Study Design and Population

An open web-based questionnaire was distributed from November 1, 2022, to January 6, 2023. The study targeted a convenience sample of women in the Netherlands who were either pregnant or had given birth in the past year and were able to read and write in Dutch. Women of any age, educational status, or BMI were eligible. Participants were recruited via social media, including Facebook advertisements, Instagram, and LinkedIn, by sharing a flyer with a link to the questionnaire (Multimedia Appendix 1). A total of 1709 women completed the questionnaire, of whom 90 (5.3%) were excluded for incomplete responses below 20% (n=77) or being both pregnant and one year postpartum (n=13), leaving data from 1619 (94.7%) women for analysis. Answers were checked for plausibility with no additional checks on bots or ungenuine respondents.

### Ethical Considerations

The study was approved by the Social Sciences Ethical Committee of Wageningen University and Research on August 23, 2022. Women checked the informed consent box at the start of the questionnaire, where information on study participation was provided, such as study activities, time investment, data handling, and purpose of the study. Study data were stored separately from names and email addresses on a secured drive of the university. As an incentive, women could participate in a lottery to win one of three €15 (US $15.90) gift vouchers.

### Questionnaire

The questionnaire, designed in Qualtrics (Qualtrics, Inc), started with a study explanation and informed consent request, followed by 65 questions distributed among 19 pages in Dutch: 32 (49.2%) multiple-choice questions, 2 (3.1%) ranking questions, 11 (16.9%) rating questions, and 20 (30.8%) open-ended questions, of which 13 (20.0%) were optional (Multimedia Appendix 2). Based on their responses, women were directed to specific questions (adaptive questioning). Completion took about 8 (IQR 6‐12) minutes. Women could navigate back to previous questions, and answers were remembered based on IP address. The questionnaire, inspired by Vasiloglou et al [31] and based on the capability, opportunity, motivation—behavior (COM-B) model [32], was developed to assess demographics, pregnancy or postpartum experience, dietary-related behaviors and their determinants, and information-seeking behaviors focusing on app use. The COM-B model served as a framework for deriving questions by defining its components, identifying relevant subtopics, and formulating specific questions accordingly. The questionnaire was pilot-tested by 5 pregnant or postpartum women on content, flow, and technical aspects, with only suggestions for slight textual adaptations.

Items questioned included age, education, work, living area, living situation (living together, not living together, or no current partner), number of children, prepregnancy BMI, planned breastfeeding, and pregnancy weeks or age of newborn in weeks. Age was calculated based on birth year and questionnaire completion year. Education was subdivided into low (primary school, vocational, or lower general secondary education), middle (higher secondary education or intermediate vocational training), and high (higher vocational education or university) based on self-reported educational levels. Hours of work were reported based on categories (equal hours, fewer hours than before pregnancy, more hours than before pregnancy, or no currently paid job). Living area was reported using categories of city and countryside. Number of children was recorded excluding the current pregnancy if pregnant. Self-reported height (cm) and weight before pregnancy (kg) were used to calculate prepregnancy BMI (kg/m^2^). Pregnancy trimester was determined based on self-reported number of weeks pregnant. Questions on pregnancy and postpartum experiences focused on overall enjoyment (rating scale) and current physical complaints.

Dietary-related experiences were addressed through subjective diet quality, and determinants of healthy dietary habits, such as considered importance of a healthy diet, nutritional knowledge, time, budget, capability to prepare and eat healthy meals, support, and enjoyment of eating. Participants were also asked about their information-seeking behaviors focusing on app use, both general and diet specific, during pregnancy and the postpartum period. Women could also specify which aspects and topics they considered important to include in a pregnancy or postpartum diet app.

### Statistical Analysis

Mean and SD were provided for continuous variables, while frequencies and percentages were presented for categorical variables. Responses to open-ended questions were inductively coded by 2 researchers independently, and codes were combined and rephrased after discussion among researchers. The most prevalent codes were synthesized in text. Pregnancy and postpartum periods were compared using chi-square tests for categorical variables and *t* tests or ANOVA for continuous variables, with significance set at *P*<.05. Analyses were conducted using RStudio (version 2023.06.1; Posit PBC).

## Results

### Characteristics of the Study Population

A total of 1619 women were included in the analysis, with an average age of 32 (SD 4) years and a mean prepregnancy BMI of 25 (SD 5) kg/m^2^. Most were highly educated (1214/1619, 75%) and lived with their partner (1570/1619, 97.0%). About half (848/1618, 52.4%) resided in a city (Table 1). Of the 842 pregnant women, half (418, 49.6%) were in their third trimester. Of the 777 postpartum women, most (517, 66.5%) gave birth less than 20 weeks ago. During pregnancy, most women (620/842, 73.6%) maintained similar work hours as before pregnancy, while postpartum women (295/777, 38.0%) worked fewer hours or the same (341/777, 43.9%) compared with before pregnancy (Table 1).

**Table 1.** General characteristics of the study population.

	Total	Pregnant women	Postpartum women	*P* value^a^
Age of woman (years; ntot=1615, npreg=842, npost=773)^b^, mean (SD)	32 (4)	32 (4)	32 (4)	.51
BMI of woman (kg/m^2^; ntot=1558, npreg=808, npost=750), mean (SD)	25 (5)	25 (5)	25 (5)	.34
Education (ntot=1619, npreg=842, npost=777), n (%)				.89
Low	5 (0.3)	2 (0.2)	3 (0.4)	
Middle	420 (25.9)	200 (23.8)	190 (24.5)	
High	1214 (75.0)	640 (76.0)	574 (73.9)	
Work (ntot=1611, npreg=838, npost=773), n (%)				<.001
Equal hours than before pregnancy	915 (56.8)	620 (73.6)	295 (38)	
Less hours than before pregnancy	441 (27.4)	100 (11.9)	341 (43.9)	
More hours than before pregnancy	18 (1.1)	10 (1.2)	8 (1)	
Currently no paid job	237 (14.6)	108 (12.8)	129 (16.6)	
Living area (ntot=1618, npreg=841, npost=777), n (%)				.03
Countryside	771 (47.6)	378 (44.9)	393 (50.6)	
City	848 (52.4)	464 (55.1)	384 (49.4)	
Trimester of pregnancy (npreg=842), n (%)				
1	—^c^	121 (14.4)	—	—
2	—	301 (35.7)	—	—
3	—	418 (59.6)	—	—
Age of newborn (weeks; npost=777), n (%)				
≤10	—	—	297 (38.2)	—
10-20	—	—	220 (28.3)	—
20-30	—	—	123 (15.8)	—
≥30	—	—	137 (17.6)	—
Partner (ntot=1619, npreg=842, npost=777), n (%)				.07
Living together	1570 (97.0)	810 (96.2)	760 (97.8)	
Not living together	21 (1.3)	16 (1.9)	5 (0.6)	
No partner	28 (1.7)	16 (1.9)	12 (1.5)	
Children^d^ (ntot=1618, npreg=842, npost=776), median (IQR)	1 (0-2)	0 (0-1)	1 (1-2)	<.001
Children^d^ (ntot=1618, npreg=842, npost=776), n (%)				<.001
0	488 (30.1)	488 (57.9)	0 (0)	
1	722 (44.6)	275 (32.7)	447 (57.5)	
2	315 (19.5)	56 (6.7)	259 (33.3)	
3	66 (4.1)	15 (7.8)	51 (6.6)	
>3	27 (1.7)	9 (1.1)	18 (2.3)	

a *P* values were calculated using chi-square tests for categorical variables and *t* tests for continuous variables.

b ntot=total number of women answering question; npreg= total number of pregnant women answering question; npost= total number of postpartum women answering question.

c Not applicable.

d Excluding current pregnancy.

### General Experiences

Women rated the postpartum period as more pleasant than pregnancy (mean 8.4, SD 1.5 vs 6.6, SD 2.1 on a 10-point scale; *P*<.001; Table 2). Common pregnancy complaints were tiredness (673/837, 80.4%), frequent toilet visits (547/837, 65.4%), heartburn (424/837, 50.7%), and back pain or pelvic instability (410/837, 49.0%). Postpartum concerns mentioned by the women in optional open answers included recovery (29/764, 3.8%), adjusting to the new situation (23/764, 3.0%) and challenging baby care (70/764, 9.2%) because of, for example, suboptimal sleep (23/764, 3.0%) or a baby who was uncomfortable and/or crying (33/764, 4.3%). Most pregnant women intended to breastfeed (737/837, 88.1%) and about half of postpartum women exclusively breastfed at the time of the survey (404/764, 52.9%).

**Table 2.** General and dietary-related experiences of women during pregnancy and postpartum period.

	Total	Pregnant women	Postpartum women	*P* value^a^
Q14 and Q17 general experience (0-10; npreg=837, npost=764)^b,c^, mean (SD)	—^d^	6.6 (2.1)	8.4 (1.5)	<.001
Q15: pregnancy complaints (n=837), n (%)^e^				
Nausea	—	288 (34.4)	—	—
Dizzy	—	168 (20.1)	—	—
More frequent toilet visits	—	547 (65.4)	—	—
Heartburn	—	424 (50.7)	—	—
Braxton Hicks (hard belly)	—	342 (40.1)	—	—
Mood swings	—	233 (27.8)	—	—
Hemorrhoids	—	97 (11.6)	—	—
Tiredness	—	673 (80.4)	—	—
Back pain or pelvic instability	—	410 (49.0)	—	—
Ligament pain	—	317 (37.8)	—	—
Fluid retention	—	109 (13.0)	—	—
Other	—	85 (10.2)	—	—
None	—	11 (1.3)	—	—
Q16: planned breastfeeding: yes (npreg=837), n (%)	—	737 (88.1)	—	—
Q19: breastfeeding past week (npost=764), n (%)				
Yes, exclusively	—	—	404 (52.9)	—
Yes, unexclusively	—	—	86 (11.3)	—
No	—	—	274 (35.9)	—
Q20 and Q25: subjective diet score (0-10; ntot=1587, npreg=830, npost=757), mean (SD)	6.9 (1.4)	6.8 (1.5)	7.0 (1.4)	.03
Q21 and Q26: importance of a healthy diet (0-10; ntot=1587, npreg=830, npost=757), mean (SD)	8.5 (1.2)	8.5 (1.2)	8.4 (1.2)	.09
Q22 and Q27: knowledge of a healthy diet during pregnancy or postpartum period (ntot=1565, npreg=820, npost=745), n (%)				<.001
A lot	887 (56.7)	533 (65)	354 (47.5)	
The basics	648 (41.4)	280 (34.1)	368 (44)	
No	30 (1.9)	7 (0.9)	23 (3.1)	
Q23 and Q28: desire to learn more on pregnancy or postpartum-specific nutrition: yes (ntot=1565, npreg=820, npost=745), n (%)	547 (35)	234 (28.5)	313 (42.0)	<.001
Q24 and Q29: information retrieval (ntot=1565, npreg=820, npost=745), n (%)^e^				
Partner	159 (10.2)	69 (8.2)	90 (11.6)	<.001
Family or friends	370 (23.6)	186 (22.7)	184 (24.7)	<.001
Health care professionals	805 (51.4)	483 (58.9)	322 (43.2)	<.001
Flyer	525 (33.5)	291 (35.5)	234 (31.4)	<.001
Website	1337 (85.4)	681 (83.0)	656 (88.1)	<.001
Apps	665 (42.5)	495 (60.4)	170 (22.8)	<.001
Other	85 (5.4)	47 (5.7)	38 (5.1)	<.001
Not applicable	77 (4.9)	13 (1.6)	64 (8.6)	<.001
Q30: who decides what to eat (ntot=1563, npreg=820, npost=745), n (%)^e^				
Me	1442 (92.3)	767 (93.5)	675 (90.6)	.008
Partner	683 (43.7)	343 (41.8)	340 (45.6)	.24
Children	20 (1.3)	11 (1.3)	9 (1.2)	.97
Housemates	9 (0.6)	7 (0.9)	2 (0.3)	.22
Q31: knowledge of food preparation (ntot=1529, npreg=803, npost=726), n (%)				.21
Yes, surely	1240 (81.1)	664 (82.7)	576 (79.3)	
Yes, probably	284 (18.6)	136 (16.9)	148 (20.4)	
No	5 (0.3)	3 (0.4)	2 (0.3)	
Q33: enough time to prepare food (ntot=1529, npreg= 803, npost= 726), n (%)				<.001
Yes	983 (64.3)	618 (77.0)	365 (50.3)	
No	519 (33.9)	169 (21.0)	350 (48.2)	
NA	27 (1.8)	16 (2.0)	11 (1.5)	
Q35: physical capability to prepare food (0-10), mean (SD)	7.1 (2.2)	6.9 (2.2)	7.3 (2.2)	.001
Q37: perception of healthy foods costs (ntot=1529, npreg= 803, npost= 726), n (%)				.62
Cheaper than unhealthy foods	68 (4.4)	32 (4.0)	36 (5.0)	
About similar	445 (29.1)	232 (28.9)	213 (29.3)	
More expensive than unhealthy food	1016 (66.4)	539 (67.1)	477 (65.7)	
Q38: influence of grocery costs, mean (SD)	4.0 (3.1)	3.8 (3)	4.2 (3.1)	.005
Q39: stress related to arranging food (ntot=1529, npreg=803, npost=726), n (%)				.008
Always	9 (0.6)	3 (0.4)	6 (0.8)	
Usually	89 (5.8)	35 (4.4)	54 (7.4)	
Sometimes	491 (32.1)	241 (30.0)	250 (34.4)	
Rarely	532 (34.8)	293 (36.5)	239 (32.9)	
Never	368 (24.1)	212 (26.4)	156 (21.5)	
Did not arrange food	40 (2.6)	19 (2.4)	21 (2.9)	
Q40: eating with family (ntot=1520, npreg=799, npost=721), n (%)				.37
Usually	1406 (92.5)	732 (91.6)	674 (93.5)	
Sometimes	88 (5.8)	51 (6.4)	37 (5.1)	
Mostly not	26 (1.7)	16 (2.0)	10 (1.4)	
Q41: enough time to eat: yes (ntot=1520, npreg=799, npost=721), n (%)	1297 (85.3)	773 (96.7)	524 (72.7)	<.001
Q43: enjoyment of eating (0-10; ntot=1520, npreg=799, npost=721)	7.1 (1.9)	7.2 (1.9)	7.0 (1.9)	.15
Q45: partner’s perceived importance of healthy eating for women (0-10; ntot=1485, npreg=781, npost=704), mean (SD)	7.7 (1.9)	7.8 (1.7)	7.5 (2)	.001
Q46: support from partner for healthy eating (0-10; ntot=1479, npreg=779, npost=700), mean (SD)	7.4 (2.1)	7.5 (2.0)	7.3 (2.2)	.07
Q47: reasons for eating (ntot=1479, npreg=779, npost=700), n (%)^e^				
Hunger	1108 (74.9)	552 (70.9)	556 (79.4)	.01
Own health	1169 (9)	631 (81.0)	538 (76.9)	.01
Child’s health	1153 (78.0)	699 (89.7)	454 (64.9)	<.001
Enjoyment	962 (65.0)	486 (62.4)	476 (68)	.16
Boredom	203 (13.7)	82 (10.5)	121 (17.3)	.001
Stress	139 (9.4)	42 (5.4)	97 (13.9)	<.001
Other	95 (6.4)	64 (8.2)	31 (4.4)	.003

a *P* values were calculated using chi-square tests for categorical variables and *t* tests for continuous variables.

b Numbering in questionnaire (Multimedia Appendix 2).

c ntot=total number of women answering question; npreg= total number of pregnant women answering question; npost= total number of postpartum women answering question.

d Not applicable.

e Multiple responses were possible.

### Eating-Related Experiences

#### Importance and Knowledge About Nutrition

Postpartum women rated their diet slightly healthier than pregnant women (mean 7.0, SD 1.4 and mean 6.8, SD 1.5 on a score of 10; *P*=.03; Table 2). Both groups valued a healthy diet equally (mean 8.5, SD 1.2 on a score of 10). More pregnant women felt knowledgeable about a healthy diet (533/820, 65.0% pregnant women vs 354/745, 47.5% postpartum women; *P*<.001), but postpartum women showed greater interest in learning about postpartum nutrition (313/745, 42.0% postpartum women vs 234/820, 28.5% pregnant women; *P*<.001). In optional open answers, pregnant women mentioned being interested in healthy pregnancy nutrition (33/820, 4.0%), nutrition for pregnancy complaints (21/820, 2.6%), and specific diets (eg, vegetarian; 15/820, 1.8%), while postpartum women focused on general healthy nutrition (64/745, 8.6%), nutrition for breastfeeding (51/745, 6.8%), and recovery (26/745, 3.5%). Information was mainly obtained from websites (1337/1565, 85.4%), HCPs (805/1565, 51.4%), and apps (665/1565, 42.5%).

#### Food Preparation

Most women primarily decided what the family ate (1442/1563, 92.3%), while 43.7% (683/1563) of partners also decided (Table 2). The majority knew how to prepare a healthy meal (1240/1529, 81.1%). Some had extensive nutrition knowledge from past experience and interest in healthy nutrition (135/1529, 8.8%) or a professional education (35/1529, 2.3%) as noted in optional open answers. More pregnant women had enough time to prepare food compared with postpartum women (618/803, 77.0% vs 365/726, 50.3%; *P*<.001), with newborn care being the main time constraint postpartum (92/726, 12.7%) as mentioned in optional open answers. Postpartum women felt more capable of food preparation than pregnant women (mean 7.3, SD 2.2 vs mean 6.9, SD 2.2 on a 10-point scale; *P*=.001), with tiredness (77/803, 9.6%) and nausea (29/803, 3.6%) being common barriers during pregnancy as mentioned in optional open answers. In both groups, stress in meal preparation was experienced rarely (532/1529, 34.8%) or sometimes (491/1529, 32.1%). Also, healthy foods were often considered more expensive (1016/1529, 66.4%), with grocery costs partly influencing purchases (mean 4.0, SD 3.1 on a 10-point scale).

#### Dietary-Related Experiences

Dinner is typically a family activity (1409/1520, 92.5%; Table 2). Almost all pregnant women felt they had enough time to eat (773/799, 96.7%) compared with 72.7% (524/721) of postpartum women (*P*<.001), with disruptions by children being the main reason for limited time mentioned in an optional open statement (71/721, 9.8%). Enjoyment of eating was similar between pregnant and postpartum women (mean 7.2, SD 1.9 and mean 7.0, SD 1.9). As mentioned in an optional open statement, pregnancy-related factors affecting enjoyment were pregnancy complaints (50/799, 6.3%), such as heartburn (17/799, 2.1%) and changed appetite (26/799, 3.3%), and a positive factor was eating with the family (15/799, 1.9%), while postpartum women cited disturbances by newborns (47/721, 6.5%) or other children (27/721, 3.7%) to negatively affect enjoyment. Partners appeared more concerned about healthy eating during pregnancy than the postpartum period (mean 7.8, SD 1.7 vs mean 7.5, SD 2.0; *P*=.001), but their support remained rather consistent (mean 7.5, SD 2.0 and mean 7.3, SD 2.2 on a 10-point scale; *P*=.07). Key reasons for eating among pregnant women were child’s health (699/779, 89.7%), their own health (631/779, 81.0%), hunger (552/779, 70.9%), and enjoyment (486/779, 62.4%), while for postpartum women, the main reasons were hunger (556/700, 79.4%), their own health (538/700, 76.9%), enjoyment (476/700, 68.0%), and child’s health (454/700, 64.9%).

### General and Dietary-Related Experiences Split on Periods of Pregnancy and Postpartum Period

Women considered the first pregnancy trimester least pleasant (mean 5.8, SD 2.7 on a 10-point scale) and the first 20 weeks postpartum most pleasant (mean 8.4, SD 1.5 on a 10-point scale). Subjective diet quality was rated lower in the first trimester (mean 6.3, SD 1.9 on a 10-point scale) and highest after 20 weeks postpartum (mean 7.1, SD 1.5 on a 10-point scale; Multimedia Appendix 3). Diet was considered most important in the first trimester (mean 9.0, SD 1.0 on a 10-point scale). Interest in learning about nutrition was highest at the start of pregnancy and early postpartum, 43.0% (49/114) and 44.1% (126/286), respectively. In the first weeks postpartum, 53.1% (152/286) of partners also decided about family meals, dropping to 38.5% (50/130) after 30 weeks. Women felt most physically capable of preparing food after 10 weeks postpartum (mean 7.8, SD 1.7 on a 10-point scale).

### Information Retrieval Using Apps

Almost all women owned a smartphone (1511/1619, 93.3%; Multimedia Appendix 4), and 37.0% (599/1619) owned a tablet. While 90.8% (713/785) of pregnant women used a pregnancy app, only 58.9% (418/710; *P*<.001) of postpartum women used a postpartum-specific app. Reasons for not using an app during pregnancy were that it was not considered necessary (24/785, 3.1%), that it was not perceived as useful (4/785, 0.5%), and that this was not the first pregnancy (8/785, 1.0%). While women during the postpartum period did not use an app because it was not considered useful (66/710, 9.3%), there was a perceived lack of a high-quality app (42/710, 5.9%), or there was a lack of time (7/710, 1.0%). Apps were discovered through searches (545/1131, 48.2%), family or friends (367/1131, 32.4%), HCPs (311/1131, 27.5%), and social media (293/1131, 25.9%). Most preferred features were reliable information, ease of use, and being freely available. In optional open answers, pregnant women also valued food safety information (225/785, 28.6%), general pregnancy information (101/785, 12.8%), and trustworthy, evidence-based information (41/785, 5.2%), while postpartum women valued healthy nutrition (191/710, 26.9%), recipes or meal plans (101/710, 14.2%), nutrition tips when breastfeeding (91/710, 12.8%), and general postpartum information (61/710, 8.6%).

## Discussion

### Principal Findings

This study provides important insights into dietary-related experiences, determinants, and information-seeking behaviors, focusing on app use, among pregnant and postpartum women. Both groups highly valued a healthy diet, though differences emerged between stages indicating a need for personalized mHealth apps. During the first pregnancy trimester, women enjoyed food the least, rated their diet lowest, but valued healthy eating the most. Postpartum women had less phase-specific nutritional knowledge but showed a stronger desire to learn about nutrition. They also reported feeling more capable of food preparation despite having less time for it compared with pregnant women. The focus on the child’s health was a major motivator for healthy eating during pregnancy, while hunger was the main motivator postpartum. Partner support was highest in the first trimester and declined over time. Women primarily obtained nutrition information from websites, HCPs, and apps. Almost all pregnant women (713/785, 90.8%) used a phase-specific app, compared with 58.9% (418/710) of postpartum women. Many women noted the apparent lack of a quality postpartum app, highlighting opportunities for improvement.

Consistent with previous research, our study showed that perspectives and experiences regarding nutrition varied between perinatal phases [13,26,33]. Therefore, when designing dietary interventions for pregnant and postpartum women, it is essential to tailor these interventions to the specific needs of each phase. Motivation, opportunity, and capability to prepare and eat healthy meals vary throughout pregnancy and the postpartum period due to several factors. Motivation to eat healthily was highest in the first trimester, driven by concern for the unborn child’s health, as also indicated by Rockliffe et al [33]. In our study, postpartum women rated their diet slightly higher than pregnant women, despite assigning less importance to healthy eating, which may suggest that they more quickly perceive their diet as sufficiently healthy.

The opportunity to eat healthily was influenced by time and social support. Our study found that pregnant women generally had more time for meal preparation and eating, while postpartum women faced time constraints mainly due to childcare priorities, which may lead them to opt for convenient, potentially unhealthy foods, as indicated by other studies [16,34]. The social environment, particularly partner support, also affects eating behavior [13,16,33,35]. In our study, partner support peaked in the first trimester but declined over time, potentially resulting in more time constraints for eating during the postpartum period. Furthermore, family dinners were common in both groups, which can provide valuable social interaction while positively influencing both maternal and children’s eating habits and weight [36-38].

The capability to prepare food and eat healthily was influenced by physical complaints and knowledge. Most mentioned complaints in our study, nausea and tiredness during pregnancy, align with other research [33,39]. Physical discomforts can affect cravings, appetite, and taste, which are important drivers of food choices, as mentioned by Groth et al [40]. Capability to prepare food was also lower in the first weeks after delivery likely because of postdelivery physical and mental complaints [41]. Furthermore, fewer postpartum women indicated having sufficient knowledge about phase-specific nutrition compared with pregnant women. Ryan et al [16] found a similar knowledge gap about healthy postpartum lifestyles among postpartum women and HCPs, likely due to limited resources [39] rather than a lack of interest, as several postpartum women in our study expressed a desire to learn more about nutrition.

To learn about nutrition, most women sought information from websites, HCPs, and mobile apps. Nonetheless, several studies reported a lack of nutritional support from HCPs (eg, due to a lack of time and knowledge) [21,26,42]. A lack of support from HCPs, as well as convenience, were important reasons for many women to use digital nutrition sources [26,43,44]. In particular, Wang et al [45] mentioned that women valued apps promoted by HCPs. In our study, 910.8% (713/785) of participants used a pregnancy app, a higher proportion than the 55% reported in Korea [46]. However, among participants in our study, only 58.9% (418/710) used postpartum-specific apps, often due to a lack of time, interest, or perceived availability. Similarly, a study from Canada found that postpartum women faced fewer nutrition resources than pregnant women [39]. This may be explained by a stronger focus on childcare over healthy maternal eating among parents as well as HCPs. In agreement, an Australian study noted that standard postpartum care prioritizes the baby’s health over the mother’s health [47]. These findings underscore the increasing role of digital tools in maternal health and the need for more focus on postpartum nutrition and improved postpartum resources.

To use a perinatal app, women in our study prioritized credible information, ease of use, and cost-free access. Ease of use was also perceived as important by Canadian pregnant women when using online sources [26]. Furthermore, credibility was crucial in the perinatal period [16,26], though conflicting advice in online resources was a common challenge during the perinatal period [42,43]. Other studies among pregnant women highlighted the importance of user-friendliness, enjoyment, and evidence-based, personalized support tailored to their situation [26,45,48,49], and postpartum women indicated preferring accurate and personalized information (eg, culturally relevant recipes) [50-52]. Comparably, women in our study ranked personalized information as the fourth priority in perinatal apps. Considering women’s preferences and the variations in their experiences across perinatal phases, tailoring dietary support in mHealth apps to their personal situations and the distinct needs of each phase appears essential.

This study used a large-scale analysis of an online questionnaire involving more than 1600 pregnant or postpartum women. However, a limitation of this study is the online distribution, which may have led to selection bias toward social media users comfortable with online surveys. While we did check for plausibility of answers, we cannot be certain that bots or ungenuine participants filled in the questionnaire. Most participants were highly educated, underrepresenting lower-educated women—a common survey bias [53,54]. If more lower-educated women had been included, research suggests potential lower motivation for and knowledge about healthy nutrition, which could have influenced our study results through lower engagement in nutrition information retrieval using mHealth apps [55-58]. Additionally, as not only lifestyle during pregnancy and the postpartum period but also the preconception period is critical for maternal and child health, preconception dietary behavior and app use should also be studied to enable intervention as early as possible within the first 1000 days.

A further limitation of this study is the primarily multiple-choice format of the questionnaire, potentially limiting response options, although we pilot-tested the instrument and included text boxes for further explanation. However, as explanations were optional in the questionnaire we might lack relevant input from a subset of respondents. Additionally, self-rated diet quality was subjective, preventing us from making connections between nutrition-related experiences, app use, and actual diet quality. In our study, we expect an overestimation of diet quality, as a study indicated that while only 29% of Dutch adults consumed at least 200 grams of vegetables daily, 41% believed they did [59]. In addition to self-reported dietary assessment or biomarker data, future studies could benefit from qualitative methods to explore detailed perspectives and experiences related to nutrition and app use during pregnancy and the postpartum period.

### Conclusions

This large questionnaire study among around 1600 pregnant or postpartum women highlights the importance of tailoring dietary support to specific perinatal phases. Physical discomfort during pregnancy and time constraints during the postpartum period emerged as key barriers to healthy eating, while maternal attention postpartum appeared to focus primarily on infant care rather than maternal health. Women considered nutritional information from HCPs to be reliable, although the dietary guidance from HCPs may be limited. These findings highlight the potential of digital tools, such as apps, to complement existing care. Participants particularly valued easy-to-use, cost-free apps that provide credible and tailored support. The apparent scarcity of postpartum nutrition resources further emphasizes the need for targeted support during this period. Insights from this study can inform the development of user-centered digital interventions to improve maternal diet (Figure 1). Future research could explore in more detail the dietary-related experiences and their determinants through interviews or focus groups and assess dietary intake more objectively using methods such as food frequency questionnaires or dietary recalls.

**Figure 1.**
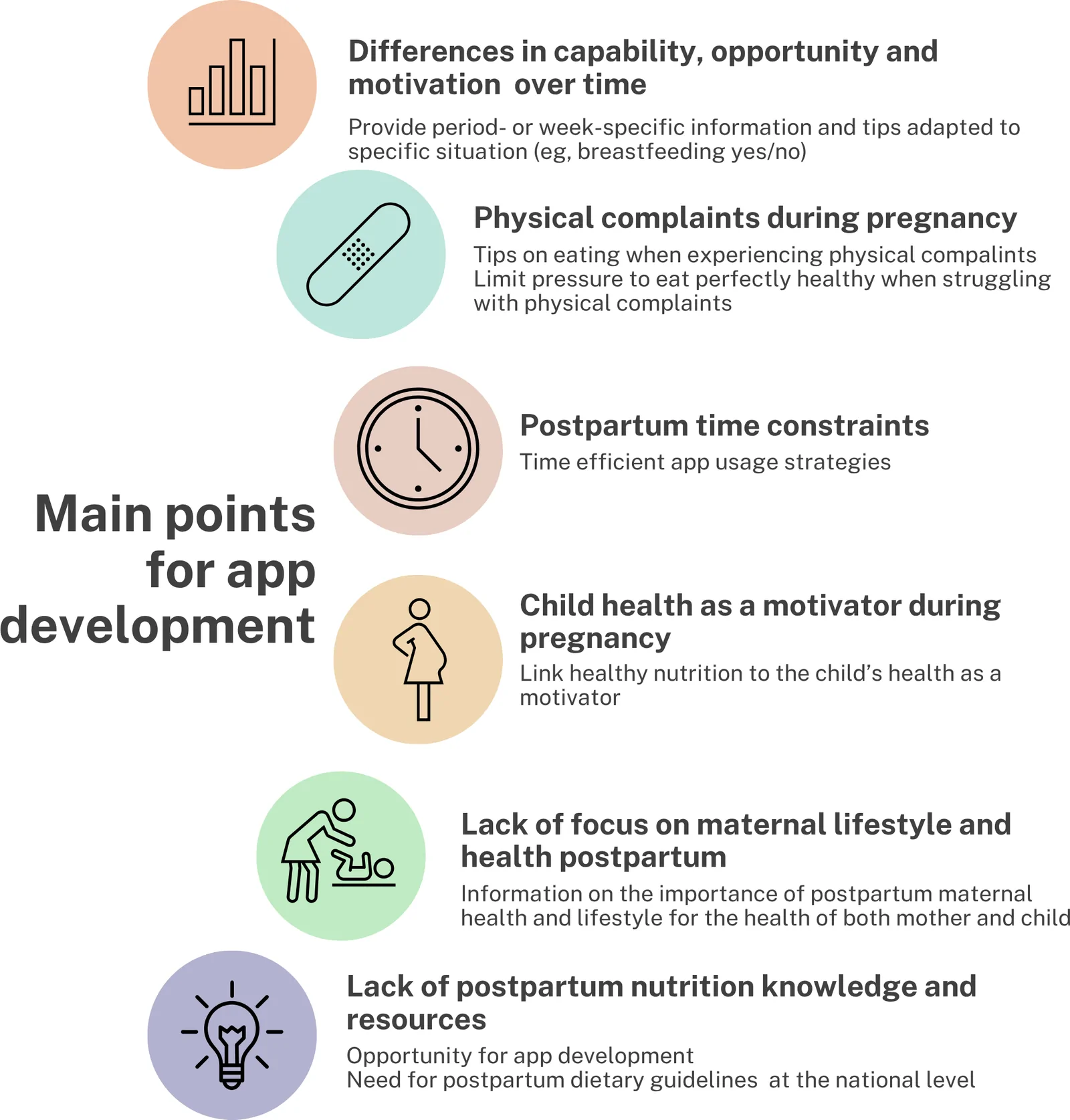
Summary of key study findings for integration into app development.

## Supplementary material

10.2196/73808 Multimedia Appendix 1 Flyer.

10.2196/73808 Multimedia Appendix 2 Questionnaire.

10.2196/73808 Multimedia Appendix 3 Results within phases.

10.2196/73808 Multimedia Appendix 4 Results apps.

## Abbreviations

COM-B: capability, opportunity, motivation–behavior
HCP: health care professional
mHealth: mobile Health
