# Supplementary material for: Differences in Dietary-Related Experiences, Determinants, and Information-Seeking Behaviors Among Pregnant and Postpartum Women: Cross-Sectional Survey Study
Source: JMIR Form Res. 2026 Sep 28;10:e73808. doi: 10.2196/73808 (PMC13618406; doi:10.2196/73808)
Supplement: Supplementary file 1 [file formative-v10-e73808-s001.pdf]

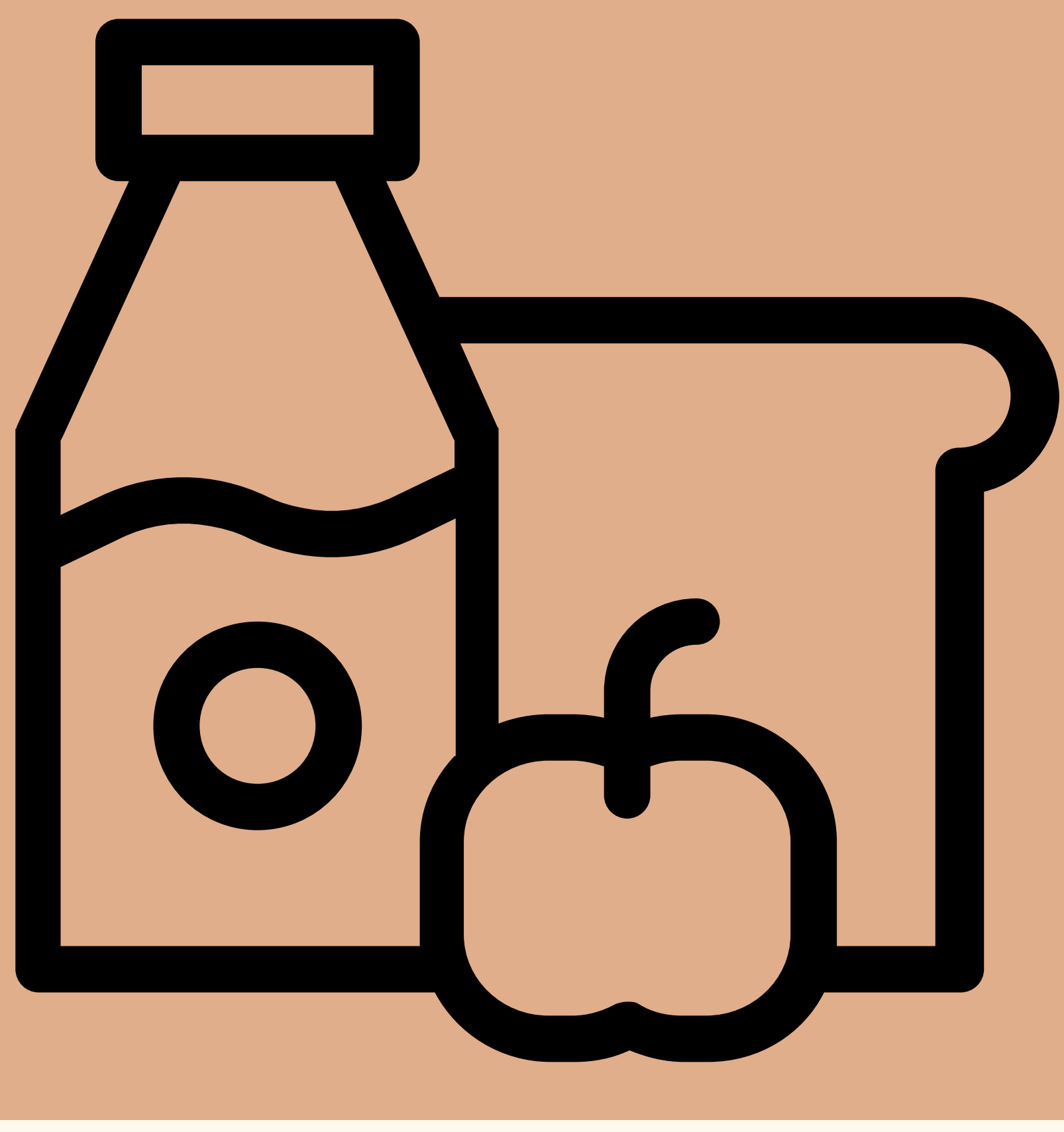

Zwanger of kort geleden bevallen van je kindje*?

*minder dan een jaar geleden

dan zoeken wij JOU!

Vul de online vragenlijst in en maak kans op een cadeaubon van €15,- van bol.com. Het invullen van de vragenlijst kost je 10 tot 15 minuten.

Wij, onderzoekers aan Wageningen Universiteit, horen graag jouw ervaringen en mening over eten in deze periode(s).

Interesse? Vul meteen de vragenlijst in!

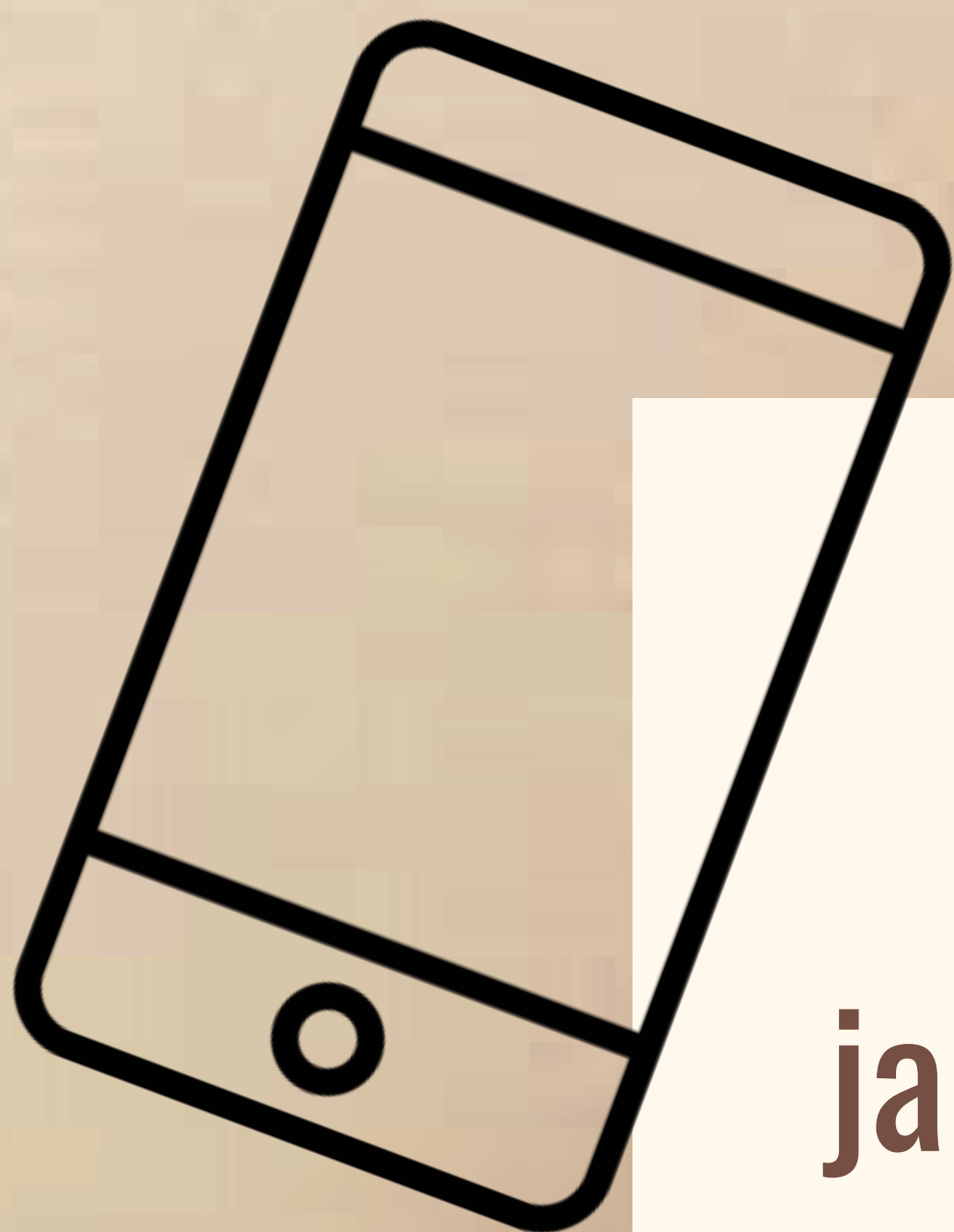

Nog vragen?
Stuur een mail naar:
janine.faessen@wur.nl

Regio Foodvalley Regio Deal
