# Supplementary material for: Differences in Dietary-Related Experiences, Determinants, and Information-Seeking Behaviors Among Pregnant and Postpartum Women: Cross-Sectional Survey Study
Source: JMIR Form Res. 2026 Sep 28;10:e73808. doi: 10.2196/73808 (PMC13618406; doi:10.2196/73808)
Supplement: Supplementary file 2 [file formative-v10-e73808-s002.docx]

**Appendix I: Questionnaire**

We, researchers from Wageningen University, are interested in your opinions and experiences as a (future) mother regarding (healthy) eating during pregnancy or the year after pregnancy! For a study at Wageningen University, we are looking for women who are currently pregnant or have given birth less than a year ago. Completing the questionnaire takes about 10 minutes. We appreciate your contribution to this study. There are no right or wrong answers. You can stop at any time by simply closing the questionnaire. By participating, you have a chance to win a €15 Bol.com gift card (3x), and you will also contribute to improved nutritional support for women during pregnancy and the year following childbirth! If you have any questions, please feel free to contact Janine: janine.faessen@wur.nl

All responses will be processed carefully, anonymously, and confidentially. The answers will only be used within this research.

o I agree to the anonymous processing of my responses by researchers at Wageningen University.

(*italic=specific questions for postpartum women,* underlined=specific questions for pregnant women)

1. Which situation applies to you?
   a. I am currently pregnant
   b. I gave birth less than a year ago
   c. I am pregnant and gave birth less than a year ago
2. How many weeks pregnant are you?

Scroll down: 0-42 weeks

3. How old is your most recently born child in weeks?

Scroll down: 0-52 weeks

4. What is your birth year? (open)
5. What is your height? (cm) (optional) (open)
6. What was your approximate weight before your pregnancy? (kg) (optional) (open)
7. What is your highest level of education completed?
   a. Primary school (elementary education, special primary education)
   b. Lower or preparatory vocational education (LBO, LTS, trade school, home economics school, and technical education (LHNO), VMBO-B (vocational or intermediate vocational education))
   c. General secondary education (MAVO, MULO, ULO, short MBO, VMBO-T (theoretical or mixed pathway))
   d. Intermediate vocational education (long MBO, vocational training pathway (BOL), vocational apprenticeship pathway (BBL), MTS, MEAO)
   e. Higher general secondary education (HAVO, pre-university education (VHBO), secondary girls' school (MMS), VWO, atheneum, gymnasium, higher civil school (HBS))
   f. Higher professional education (HBO, teacher training (PABO), conservatory, MO certificates, new style colleges, HTS, HEAO)
   g. Scientific education (WO, university, old-style technical/economic college)
8. Are you currently employed? (paid work)
   a. Yes, the same number of hours as before pregnancy
   b. Yes, fewer hours than before pregnancy
   c. Yes, more hours than before pregnancy
   d. No, I am currently not working
9. In what province do you live?
   a. Groningen
   b. Friesland
   c. Drenthe
   d. Overijssel
   e. Flevoland
   f. Gelderland
   g. Utrecht
   h. Noord-Holland
   i. Zuid-Holland
   j. Zeeland
   k. Noord-Brabant
   l. Limburg
10. In what type of area do you live?
    a. Urban area
    b. Non-urban area
11. Do you currently have a partner?
    a. Yes, I live with my partner
    b. Yes, but we do not live together
    c. No
12. *How many children do you have? (open)*
13. How many children you do have? (excluding current pregnancy) (open)

The next questions are about your pregnancy experiences

14. How have you experienced your pregnancy in the past month? (0=unpleasant, 10 =very pleasant)

Rating scale 0-10

15. Which pregnancy symptoms have you experienced in the past week? (multiple answers possible)
    a. Nausea
    b. Dizziness
    c. Frequent urination
    d. Heartburn
    e. Hard belly
    f. Mood swings
    g. Hemorrhoids
    h. Fatigue
    i. Back pain or pelvic instability
    j. Round ligament pain
    k. Fluid retention
    l. Other, namely …..
    m. None
16. Do you plan to breastfeed after pregnancy?
    a. Yes
    b. No

*The next questions are about your experiences in the period after pregnancy*

17. *How have you experienced the past month with your newborn? (0=unpleasant, 10=very pleasant)*

*Rating scale 0-10*

18. *Would you like to elaborate on this further? (open) (optional)*
19. *Did you breastfeed your child in the past week?*
    a. *Yes, exclusively*
    b. *Yes, partially*
    c. *No*

The following questions are about your experiences with nutrition during your pregnancy

20. What rating would you give your nutritional intake over the past month? (0=unhealthy, 10=healthy)

Rating scale 0-10

21. How important do you consider healthy nutrition during pregnancy? (0=not important, 10= very important)

Rating scale 0-10

22. Do you think you have sufficient knowledge about healthy nutrition during pregnancy?
    a. Yes, quite a lot of knowledge
    b. Yes, I know the basics
    c. No
23. Would you like to learn more about healthy nutrition during pregnancy?
    a. Yes, for example about ….
    b. No
24. Where do you get information from if you have questions about nutrition during pregnancy? (multiple answers possible)
    a. Partner
    b. Family/friends
    c. Healthcare professionals
    d. Brochures/books
    e. Websites
    f. Apps
    g. Other, namely ….
    h. I have no questions

*The following questions are about your experiences with nutrition in the year following pregnancy.*

25. *What rating would you give your nutritional intake over the past month? (0=unhealthy, 10=healthy)*

*Rating scale 0-10*

26. *How important do you consider healthy nutrition after pregnancy? (0=not important, 10= very important)*

*Rating scale 0-10*

27. *Do you think you have sufficient knowledge about healthy nutrition after pregnancy?*
    a. *Yes, quite a lot of knowledge*
    b. *Yes, I know the basics*
    c. *No*
28. *Would you like to learn more about healthy nutrition after pregnancy?*
    a. *Yes, namely …*
    b. *No*
29. *Where do you get information from if you have questions about nutrition after pregnancy?(multiple answers possible)*
    a. *Partner*
    b. *Family/friends*
    c. *Healthcare professionals*
    d. *Brochures/books*
    e. *Websites*
    f. *Apps*
    g. *Other, namely ….*
    h. *I have no questions*

The following questions are about your experience with buying and preparing food, as well as your eating experiences over the past month.

30. Who primarily decided what was eaten over the past month? (multiple answers possible)
    a. I
    b. My partner
    c. My children
    d. Other housemates
    e. Other, namely: …
31. Do you feel you have sufficient knowledge to buy and prepare a healthy meal?
    a. Yes, definitely
    b. Yes, probably
    c. No
32. Would you like to explain your answer? (open) (optional)
33. Do you feel that you had enough time to buy and prepare food over the past month?
    a. Yes
    b. No
    c. Not applicable
34. Would you like to explain your answer? (open) (optional)
35. To what extent did you feel physically able to manage meal preparation over the past month? (0= not at all, 10=completely)

Rating scale 0-10

36. Would you like to explain your answer? (open) (optional)
37. What is your perception of the cost of healthy eating?
    a. Cheaper than unhealthy eating
    b. About the same as unhealthy eating
    c. More expensive than unhealthy eating
38. To what extent was your choice of groceries influenced by the (rising) cost of groceries over the past month? (0=not at all, 10=very strongly)

Rating scale 0-10

39. Did arranging (healthy) meals cause you stress over the past month?
    a. Always
    b. Often
    c. Sometimes
    d. Rarely
    e. Never
    f. I did not arrange meals over the past month
40. Did you have meals with your family over the past month?
    a. Yes, most of the time
    b. Yes, sometimes
    c. No, not usually
41. Do you feel that you had enough time to eat over the past month?
    a. Yes
    b. No
42. Would you like to explain your answer? (open) (optional)
43. How enjoyable did you find eating moments over the past month? (0=not enjoyable, 10=very enjoyable)

Rating scale 0-10

44. Would you like to explain your answer? (open) (optional)
45. How important does your partner think it is for you to eat healthily? (0=not important, 10=very important)

Rating scale 0-10

46. To what extent has your partner supported and motivated you to eat healthily over the past month? (0=not at all, 10 = a great deal)

Rating scale 0-10

47. What were the main reasons for eating over the past month? (multiple answers possible)
    a. To satisfy hunger
    b. To get important nutrients for myself
    c. To get important nutrients for my child
    d. To enjoy taste food
    e. To combat boredom
    f. To alleviate stress
    g. Other, namely …

The following questions are about app use on a mobile phone or tablet.

48. Do you have a smartphone and/or tablet? (multiple answers possible)
    a. Yes, smartphone
    b. Yes, tablet
    c. No
49. What do you currently use your smartphone for? (multiple answers possible)
    a. (video) calling/SMS
    b. WhatsApp
    c. Email
    d. Social media
    e. Games
    f. Internet browsing
    g. Taking photos
    h. Other, namely …
50. What do you currently use your tablet for? (multiple answers possible)
    a. (video) calling
    b. WhatsApp
    c. Email
    d. Social media
    e. Games
    f. Internet browsing
    g. Taking photos
    h. Other, namely …

The following questions are about apps specifically for use during pregnancy.

51. Are you currently using or have used pregnancy apps?
    a. Yes, namely …
    b. No
52. For what purposes do/did you use these apps? (open)
53. Why do/did you not use these apps? (open) (optional)
54. How did you come across these pregnancy app(s)? (multiple answers possible)
    a. Social media
    b. Through advertisements
    c. Through family/friends/acquaintances
    d. Through a healthcare professional
    e. Searched for them yourself
    f. I don’t remember
    g. Other, namely: …

*The following questions are about apps specifically for use during the period after pregnancy (consider apps about motherhood, postpartum recovery, or the baby).*

55. *Are you currently using or have you used apps about the year after pregnancy?*
    a. *Yes, namely …*
    b. *No*
56. *For what purposes do/did you use these apps? (open)*
57. *Why do/did you not use these apps? (open) (optional)*
58. *How did you come across these app(s) about the year after pregnancy? (multiple answers possible)*
    a. *Social media*
    b. *Through advertisements*
    c. *Through family/friends/acquaintances*
    d. *Through a healthcare professional*
    e. *Searched for them yourself*
    f. *I don’t remember*
    g. *Other, namely: …*

The following questions are about what you consider important in an app.

59. What do you consider important topics in a (nutrition) app during pregnancy? (open)
60. Please rank the following options for a (nutrition) app during pregnancy from most important (1) to least important (9) by dragging them up or down.
    a. Easy to use
    b. Free
    c. Track nutritional intake
    d. Game element
    e. Reliable information
    f. Minimal text with use of illustrations and/or videos
    g. Chat with other women
    h. Contact information for healthcare professionals
    i. Advice tailored to your specific situation (personalized)
61. What feature for the app do you find important that was not mentioned in the previous question? (open) (optional)
62. *What do you consider important topics in a (nutrition) app after pregnancy? (open)*
63. *Please rank the following options for a (nutrition) app after pregnancy from most important (1) to least important (9) by dragging them up or down.*
    a. *Easy to use*
    b. *Free*
    c. *Track nutritional intake*
    d. *Game element*
    e. *Reliable information*
    f. *Minimal text with use of illustrations and/or videos*
    g. *Chat with other women*
    h. *Contact information for healthcare professionals*
    i. *Advice tailored to your specific situation (personalized)*
64. *What feature for the app do you find important that was not mentioned in the previous question? (open) (optional)*
65. Do you have any additional remarks? (open) (optional)
66. Thank you very much for completing this questionnaire! If you would like to have a chance to win a gift voucher, please enter your email address and/or phone number below. Your details will only be used for the lottery. We will contact the winners!
    a. yes, my email address and/or phone number is: …
    b. no
67. It would greatly help if you would be willing to participate in a follow-up study on nutrition for (future) mothers. Would you be interested in helping us with this? We will then contact you to provide further details, after which you can decide whether or not you want to participate in the follow-up study.
    a. Yes, I am interested in the follow-up study, any comments: …
    b. No, I do not want to be contacted for potential follow-up research
68. Please provide your email address and/or phone number again (open).

Your details will only be used to contact you about possible follow-up research.
