# Supplementary material for: Differences in Dietary-Related Experiences, Determinants, and Information-Seeking Behaviors Among Pregnant and Postpartum Women: Cross-Sectional Survey Study
Source: JMIR Form Res. 2026 Sep 28;10:e73808. doi: 10.2196/73808 (PMC13618406; doi:10.2196/73808)
Supplement: Supplementary file 3 [file formative-v10-e73808-s003.docx]

**Appendix II: Experiences of women during periods of pregnancy and postpartum.**

| Experiences eating | | | | | | | | |
| --- | --- | --- | --- | --- | --- | --- | --- | --- |
|  | Pregnancy | | | Postpartum (weeks) | | | |  |
|  | T1 | T2 | T3 | ≤10 | >10 and ≤20 | >20 and ≤30 | 30> | P-value^a^ |
| Q14. General experience (0-10), mean ±sd  (nT1=118, nT2=301, nT3=416, nP1=292, nP2=216, nP3=121, nP4=135) | 5.8 ±2.7 | 6.8 ±1.9 | 6.8 ±2.0 | 8.4 ±1.4 | 8.4 ±1.5 | 8.2±1.8 | 8.3±1.4 | **<0.001** |
| Q15/17. Pregnancy complaints, n (%) ^b^  (nT1=118, nT2=301, nT3=416)  Nausea  Dizzy  Urinate  Heartburn  Braxton Hicks (hard belly)  Mood swings  Haemorrhoids  Tired  Backpain/pelvic instability  Ligament pain  Fluid retention  Other  None | 91 (77.1)  37 (31.4)  68 (57.6)  30 (25.4)  4 (3.4)  42 (35.6)  3 (2.5)  105 (89.0)  27 (22.9)  23 (19.5)  9 (7.6)  12 (10.2)  3 (2.5) | 98 (33)  66 (22)  184 (61)  109 (36)  73 (24)  93 (31)  22 (7)  231 (77)  135 (45)  126 (42)  14 (5)  28 (9)  6 (2) | 99 (23,8)  65 (15.6)  294 (70.7)  284 (68.3)  263 (63.2)  98 (23.6)  72 (17.3)  336 (80.8)  247 (59.4)  166 (39.9)  85 (20.4)  45 (10.8)  2 (0.5) |  |  |  |  | <0.001  0.001  0.003  <0.001  <0.001  0.016  <0.001  0.064  <0.001  <0.001  <0.001  0.811  0.100 |
| Q16. Planned breastfeeding (n, %) yes  (nT1=118, nT2=301, nT3=416) | 103 (87.3) | 260 (86.4) | 373 (89.7) |  |  |  |  | 0.387 |
| Q19. Breastfeeding past week, n (%)  (nP1=292, nP2=216, nP3=121, nP4=135)  Exclusively  Unexclusively  No |  |  |  | 187 (64.0)  39 (13.4)  66 (22.6) | 122 (56.5)  14 (6.5)  80 (37.0) | 56 (46.3)  10 (8.3)  55 (45.5) | 39 (28.9)  23 (17.0)  73 (54.1) | <0.001 |
| Q20/25. Subjective score diet (0-10), mean ±sd (nT1=114, nT2=293, nT3=411, nP1=292, nP2=216, nP3=121, nP4=135) | 6.3±1.9 | 6.7±1.4 | 7.1±1.3 | 7.0±1.4 | 6.8±1.4 | 7.1±1.5 | 7.1±1.4 | <0.001 |
| Q21/26. Importance healthy diet (0-10),  mean ±sd (nT1=114, nT2=293, nT3=411, nP1=292, nP2=216, nP3=121, nP4=135) | 9.0±1.0 | 8.5±1.1 | 8.4±1.2 | 8.4±1.1 | 8.5±1.2 | 8.4±1.3 | 8.4±1.3 | <0.001 |
| Q22/27. Knowledge healthy diet, n (%)  (nT1=114, nT2=293, nT3=411, nP1=286, nP2=211, nP3=118, nP4=130)  A lot  The basics  No | 68 (59.6)  46 (40.4)  0 (0.0) | 187 (63.8)  103 (35.2)  3 (1.0) | 277 (67.4)  130 (31.6)  4 (1.0) | 129 (45.1)  150 (52.4)  7 (2.4) | 100 (47.4)  103 (48.8)  8 (3.8) | 59 (50.0)  56 (47.5)  3 (2.5) | 66 (50.8)  59 (45.4)  5 (3.8) | <0.001 |
| Q23/28. Desire to learn more, n (%) yes  (nT1=114, nT2=293, nT3=411, nP1=286, nP2=211, nP3=118, nP4=130) | 49 (43.0) | 81 (27.6) | 102 (24.8) | 126 (44.1) | 88 (41.7) | 47 (39.8) | 52 (40.0) | <0.001 |
| Q24/29. Information retrieval, n (%) ^b^  (nT1=114, nT2=293, nT3=411, nP1=286, nP2=211, nP3=118, nP4=130)  Partner  Family friends  HCP  Flyer  Website  Apps  Other  NA | 12 (9.9)  27 (22.3)  60 (49.6)  44 (36.4)  101 (83.5)  67 (55,4)  5 (4.1)  0 (0.0) | 17 (5.6)  58 (19.3)  161 (53.5)  105 (34.9)  238 (79.1)  186 (61.8)  16 (5.3)  5 (1.7) | 40 (9.6)  101 (24.2)  261 (62.4)  142 (34.0)  340 (81.3)  242 (57.9)  26 (6.2)  8 (1.9) | 35 (12.2)  62 (21.7)  137 (47.9)  84 (29.4)  262 (91.6)  63 (22.0)  6 (2.1)  24 (8.4) | 27 (12.8)  66 (31.3)  83 (39.3)  63 (30.0)  185 (87.7)  50 (23.7)  13 (6.2)  16 (7.6) | 7 (5.9)  26 (22.0)  49 (41.5)  41 (34.7)  96 (81.4)  23 (19.5)  10 (8.5)  14 (11.9) | 21 (16.2)  30 (23.1)  53 (40.8)  46 (35.4)  113 (86.9)  34 (26.2)  9 (6.9)  10 (7.7) | 0.015  0.128  <0.001  0.419  0.068  <0.001  0.123  <0.001 |
| Q30. Who decides what to eat, n (%) ^b^  (nT1=114, nT2=293, nT3=411, nP1=286, nP2=211, nP3=118, nP4=130)  Me  Partner  Children  Housemates | 106 (87.6)  47 (38.8)  0 (0.0)  1 (0.8) | 268 (89.0)  122 (40.5)  2 (0.7)  4 (1.3) | 392 (93.8)  173 (41.4)  9 (2.2)  2 (0.5) | 247 (86.4)  152 (53.1)  5 (1.7)  1 (0.3) | 193 (91.5)  93 (44.1)  2 (0.9)  1 (0.5) | 110 (93.2)  45 (38.1)  2 (1.7)  0 (0.0) | 125 (96.2)  50 (38.5)  0 (0.0)  0 (0.0) | 0.001  0.027  0.258  0.518 |
| Q31. Enough knowledge prepare food, n (%)  (nT1=111, nT2=286, nT3=404, nP1=280, nP2=207, nP3=112, nP4=127)  Yes, surely  Yes, probably  No | 88 (79.3)  23 (20.7)  0 (0.0) | 232 (81.1)  52 (18.2)  2 (0.7) | 342 (84.7)  61 (15.1)  1 (0.2) | 227 (81.1)  53 (18.9)  0 (0.0) | 159 (76.8)  47 (22.7)  1 (0.5) | 93 (83.0)  19 (17.0)  0 (0.0) | 97 (76.4)  29 (22.8)  1 (0.8) | <0.001 |
| Q33. Enough time to prepare food, n (%)  (nT1=111, nT2=286, nT3=404, nP1=280, nP2=207, nP3=112, nP4=127)  Yes  No  NA | 81 (73.0)  27 (24.3)  3 (2.7) | 212 (74.1)  70 (24.5)  4 (1.4) | 323 (80.0)  72 (17.8)  9 (2.2) | 133 (47.5)  141 (50.4)  6 (2.1) | 110 (53.1)  94 (45.4)  3 (1.4) | 56 (50.0)  54 (48.2)  2 (1.8) | 66 (52.0)  61 (48.0)  0 (0.0) | <0.001 |
| Q35. Physical capability prepare food,  mean ±sd  (nT1=111, nT2=286, nT3=404, nP1=280, nP2=207, nP3=112, nP4=127) | 6.3±2.6 | 6.9±2.3 | 7.1±2.0 | 6.5±2.5 | 7.8±1.7 | 7.9±1.8 | 7.9±1.7 | <0.001 |
| Q37. Perception costs healthy foods, n (%)  (nT1=111, nT2=286, nT3=404, nP1=280, nP2=207, nP3=112, nP4=127)  Cheaper than unhealthy foods  About similar  More expensive than unhealthy food | 3 (2.7)  34 (30.6)  74 (66.7) | 13 (4.5)  80 (28.0)  193 (67.5) | 16 (4.0)  118 (29.2)  270 (66.8) | 9 (3.2)  84 (30.0)  187 (66.8) | 16 (7.7)  60 (29.0)  131 (63.3) | 5 (4.5)  32 (28.6)  75 (67.0) | 6 (4.7)  37 (29.1)  84 (66.1) | 0.812 |
| Q38. Influence costs groceries (0-10),  mean ±sd  (nT1=111, nT2=286, nT3=404, nP1=280, nP2=207, nP3=112, nP4=127) | 2.8±2.8 | 3.9±3.0 | 4.0±3.0 | 3.9±3.0 | 4.5±3.1 | 4.2±3.1 | 4.6±3.1 | <0.001 |
| Q39. Stress arranging food, n (%)  (nT1=111, nT2=286, nT3=404, nP1=280, nP2=207, nP3=112, nP4=127)  Always  Usually  Sometimes  Rarely  Never  Did not arrange food | 2 (1.8)  7 (6.3)  35 (31.5)  35 (31.5)  28 (25.2)  4 (3.6) | 1 (0.3)  13 (4.5)  91 (31.8)  111 (38.8)  62 (21.7)  8 (2.8) | 0 (0)  15 (3.7)  114 (28.2)  147 (36.4)  121 (30.0)  7 (1.7) | 6 (2.1)  12 (4.3)  92 (32.9)  85 (30.4)  68 (24.3)  17 (6.1) | 0 (0.0)  17 (82)  74 (35.7)  71 (34.3)  42 (20.3)  3 (1.4) | 0 (0.0)  12 (10.7)  31 (27.7)  43 (38.4)  25 (22.3)  1 (0.9) | 0 (0.0)  13 (10.2)  53 (41.7)  30 (31.5)  21 (16.5)  0 (0.0) | <0.001 |
| Q40. Eat with family, n (%)  (nT1=110, nT2=286, nT3=401, nP1=279, nP2=205, nP3=111, nP4=126)  Yes, usually  Yes, sometimes  No, mostly not | 97 (88.2)  9 (8.2)  4 (3.6) | 260 (90.9)  20 (7.0)  6 (2.1) | 373 (93.0)  22 (5.5)  6 (1.5) | 264 (94.6)  13 (4.7)  2 (0.7) | 187 (91.2)  15 (7.3)  3 (1.5) | 106 (95.5)  5 (4.5)  0 (0.0) | 117 (92.9)  4 (3.2)  5 (4.0) | 0.202 |
| Q41. Enough time to eat, n (%) yes  (nT1=110, nT2=286, nT3=401, nP1=279, nP2=205, nP3=111, nP4=126) | 104 (94.5) | 277 (96.9) | 390 (97.3) | 201 (72) | 147 (72) | 86 (78) | 90 (71) | <0.001 |
| Q43. Enjoyment eating (0-10), mean ±sd  (nT1=110, nT2=286, nT3=401, nP1=279, nP2=205, nP3=111, nP4=126) | 6.3±2.5 | 7.0±2.0 | 7.5±1.6 | 7.0±1.9 | 6.9±1.9 | 6.9±2.0 | 7.4±1.5 | <0.001 |
| Q45. Perceived importance food partner (0-10), mean ±sd  (nT1=110, nT2=286, nT3=401, nP1=279, nP2=205, nP3=111, nP4=126) | 8.1±2.0 | 7.7±1.6 | 7.8±1.7 | 7.7±1.8 | 7.4±2.1 | 7.3±2.1 | 7.3±2.2 | 0.006 |
| Q46. Support partner healthy eating (0-10), mean ±sd  (nT1=110, nT2=286, nT3=401, nP1=279, nP2=205, nP3=111, nP4=126) | 7.9±2.0 | 7.4±2.0 | 7.5±2.0 | 7.5±2.1 | 7.3±2.2 | 7.3±2.1 | 7.1±2.5 | 0.105 |
| Q47. Reason to eat, n (%) ^b^  (nT1=110, nT2=286, nT3=401, nP1=279, nP2=205, nP3=111, nP4=126)  Hunger  Health self  Health child  Enjoy  Boredom  Stress  Other | 75 (68.2)  90 (81.8)  88 (80)  51 (46.4)  7 (6.4)  8 (7.3)  18 (16.4) | 204 (71.3)  220 (76.9)  250 (87.4)  169 (59.1)  21 (7.3)  16 (5.6)  24 (8.4) | 271 (67.6)  320 (79.8)  360 (89.8)  266 (66.3)  53 (13.2)  18 (4.5)  22 (5.5) | 209 (74.9)  218 (78.1)  212 (76.0)  181 (64.9)  37 (13.3)  23 (8.2)  11 (3.9) | 162 (79.0)  150 (73.2)  118 (57.6)  136 (66.3)  42 (20.5)  28 (13.7)  10 (4.9) | 85 (76.6)  78 (70.3)  55 (49.5)  66 (59.5)  19 (17.1)  23 (20.7)  6 (5.4) | 100 (79.4)  92 (73.0)  69 (54.8)  93 (73.8)  23 (18.3)  23 (18.3)  4 (3.2) | <0.001 |

^a^ p-value from chi-square tests for categorical variables and ANOVA-tests for continuous variables, ^b^ more possible answer
