# Supplementary material for: Differences in Dietary-Related Experiences, Determinants, and Information-Seeking Behaviors Among Pregnant and Postpartum Women: Cross-Sectional Survey Study
Source: JMIR Form Res. 2026 Sep 28;10:e73808. doi: 10.2196/73808 (PMC13618406; doi:10.2196/73808)
Supplement: Supplementary file 4 [file formative-v10-e73808-s004.docx]

**Appendix III: Smartphone, tablet and app use.**

|  | Total | Pregnant women | Women postpartum | p-value^a^ |
| --- | --- | --- | --- | --- |
| Own a smartphone, n (%)  (ntot=1619, npreg=842, npost=777) | 1511 (93.3) | 796 (94.5) | 715 (92.0) | 0.054 |
| Usage smartphone, n (%) ^b^  (ntot=1511, npreg=796, npost=715)  Call/text  Whatsapp  Email  Social media  Games  Internet  Camera  Other | 1283 (84.9)  1506 (99.7)  1392 (92.1)  1443 (95.5)  557 (36.9)  1352 (89.5)  1433 (94.8)  48 (3.2) | 668 (83.9)  793 (99.6)  726 (91.2)  762 (95.7)  321 (40.3)  720 (90.5)  738 (92.7)  26 (3.3) | 615 (86.0)  713 (99.7)  666 (93.1)  681 (95.2)  236 (33.0)  632 (88.4)  695 (97.2)  22 (3.1) | 0.288  1.000  0.193  0.742  0.004  0.223  <0.001  0.950 |
| Own a tablet, n (%)  (ntot=1619, npreg=842, npost=777) | 599 (37.0) | 309 (36.7) | 290 (37.3) | 0.835 |
| Usage tablet, n (%) ^b^  (ntot=599, npreg=309, npost=290)  Call/text  Whatsapp  Email  Social media  Games  Internet  Camera  Other | 29 (4.8)  5 (0.8)  133 (22.2)  121 (20.2)  193 (32.2)  328 (54.8)  10 (1.7)  262 (43.7) | 16 (5.2)  3 (1.0)  62 (20.1)  59 (19.1)  94 (30.4)  170 (55.0)  4 (1.3)  137 (44.3) | 13 (4.5)  2 (0.7)  71 (24.5)  62 (21.4)  99 (34.1)  158 (54.5)  6 (2.1)  125 (43.1) | 0.837  1.000  0.229  0.552  0.376  0.961  0.674  0.825 |
| Usage apps (pregnancy/ postpartum), n (%) yes  (ntot=1495, npreg=785, npost=710) | 1131 (75.7) | 713 (90.8) | 418 (58.9) | <0.001 |
| Know apps via: n (%) ^b^  (ntot=1131, npreg=713, npost=418)  Social media  Ads  Family  Hcp  Search  Don’t know  Other | 293 (25.9)  68 (6.0)  367 (32.4)  311 (27.5)  545 (48.2)  102 (8.9)  41 (3.4) | 182 (25.5)  41 (5.8)  241 (33.8)  218 (30.6)  346 (48.5)  65 (9.1)  21 (2.9) | 111 (26.6)  27 (6.5)  126 (30.1)  93 (22.2)  199 (47.6)  36 (8.6)  17 (4.1) | 0.756  0.723  0.229  0.003  0.813  0.858  0.401 |
| Rank importance aspects/features app | 1. Reliable info 2. Easy to use 3. No costs 4. Personalised 5. Tracking diet 6. Little text, more illustrations 7. Contact HCP 8. Chat women 9. Game | 1. Reliable info 2. Easy to use 3. No costs 4. Personalised 5. Tracking diet 6. Little text, more illustrations 7. Contact HCP 8. Game 9. Chat women | 1. Reliable info 2. Easy to use 3. No costs 4. Personalised 5. Tracking diet 6. Little text, more illustrations 7. Contact HCP 8. Chat women 9. Game |  |

^a^ p-value from chi-square tests for categorical variables and ANOVA-tests for continuous variables, ^b^ more possible answers
